# REAL-Select: Full-Length Antibody Display and Library Screening by Surface Capture on Yeast Cells

**DOI:** 10.1371/journal.pone.0114887

**Published:** 2014-12-12

**Authors:** Laura Rhiel, Simon Krah, Ralf Günther, Stefan Becker, Harald Kolmar, Björn Hock

**Affiliations:** 1 Protein Engineering and Antibody Technologies, Merck Serono, Merck KGaA, Darmstadt, Germany; 2 Institute for Organic Chemistry and Biochemistry, Technische Universität Darmstadt, Darmstadt, Germany; Louisiana State University, United States of America

## Abstract

We describe a novel approach named REAL-Select for the non-covalent display of IgG-molecules on the surface of yeast cells for the purpose of antibody engineering and selection. It relies on the capture of secreted native full-length antibodies on the cell surface via binding to an externally immobilized ZZ domain, which tightly binds antibody Fc. It is beneficial for high-throughput screening of yeast-displayed IgG-libraries during antibody discovery and development. In a model experiment, antibody-displaying yeast cells were isolated from a 1∶1,000,000 mixture with control cells confirming the maintenance of genotype-phenotype linkage. Antibodies with improved binding characteristics were obtained by affinity maturation using REAL-Select, demonstrating the ability of this system to display antibodies in their native form and to detect subtle changes in affinity by flow cytometry. The biotinylation of the cell surface followed by functionalization with a streptavidin-ZZ fusion protein is an approach that is independent of the genetic background of the antibody-producing host and therefore can be expected to be compatible with other eukaryotic expression hosts such as *P. pastoris* or mammalian cells.

## Introduction

Yeast surface display is a powerful tool for library selection and protein engineering especially in the field of antibody discovery and development [1], [2]. During biological drug development, it can contribute to the fast identification and optimization of human antibodies and drug candidates due to its compatibility with fluorescence-activated cell sorting and its applicability to high-throughput screening [3], [4]. *Saccharomyces cerevisiae* is an approved host for the synthesis and secretion of complex proteins such as antibody scFv [5] and Fab fragments [6]. Current yeast display technologies take advantage of the presence of naturally occurring GPI (Glycosylphosphatidylinositol) anchor proteins in the cell wall that can function as an anchor unit for the display of recombinant proteins, *e. g.* antibody fragments [7], [8]. In this set-up the protein of interest is fused to the *C*- or *N*-terminal part of the respective anchor protein, directing the recombinant fusion protein to the outer cell surface [9], [10]. In recent years, several different anchor proteins have been identified that facilitate the display of foreign proteins on yeast cells [11], [12], but the most prominent one is the Aga2p-dependend surface-display that was invented by Boder and Wittrup in 1997 [9]. This system has previously been used to engineer various antibody formats such as scFv-fragments [13], [14], Fab-fragments [15], [16], Fcabs [17], [18], and llama single domain antibody VHH-fragments [19] aimed at isolating clones with improved properties. The display of full-length IgG-molecules has not yet been described using this technology.

For the cell surface display of whole IgG-molecules on yeast cells, an alternative approach was described recently that relies on secretion of antibodies followed by capture to the surface by binding to a capturing agent. In an elegant application, Rakestraw and coworkers synthesized biotinylated antibodies by extending the CH3 domain with a biotin ligase recognition sequence and co-expression of a biotin ligase in *S. cerevisiae* followed by antibody capture to surface-immobilized avidin [20]. Since this yeast display method relies on an antibody fusion to a small peptide rather than a large cell-wall protein and uses the normal secretion pathway for folding, glycosylation, and quality check, complex protein units such as full-length IgG can be displayed on the cell surface.

**Figure 1:**
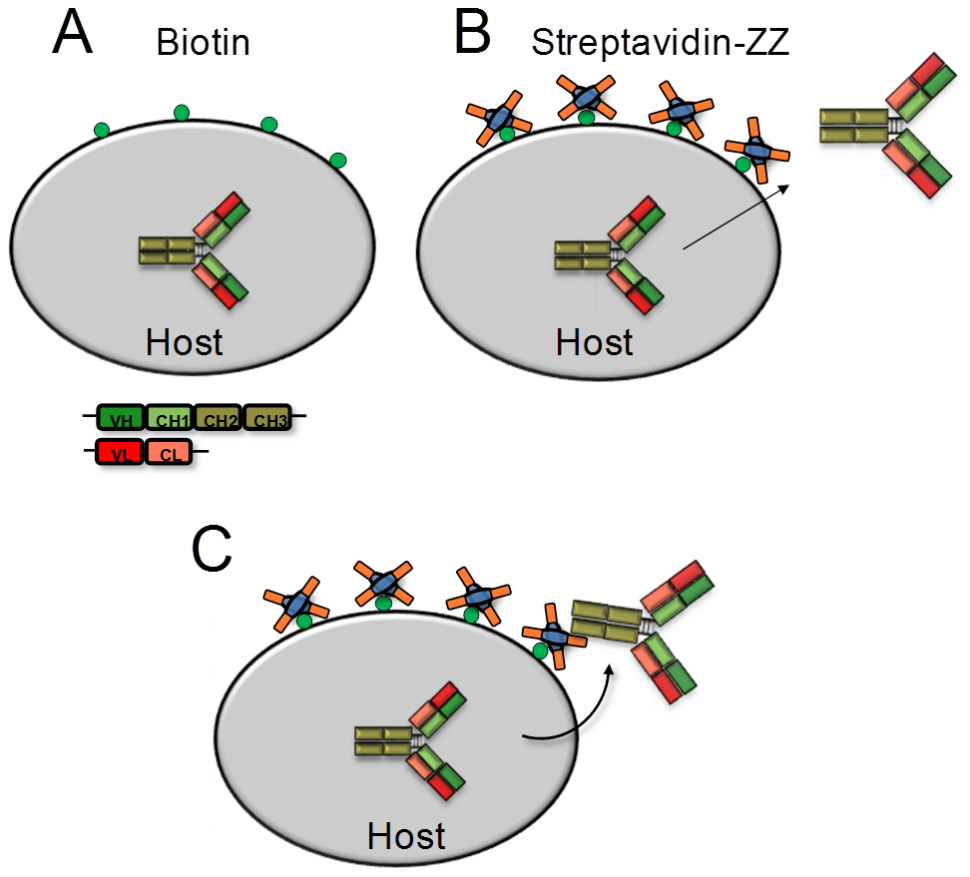
Schematic illustration of host cells modified by REAL-Select for the purpose of endogeneous antibody cell surface display. Host cells carrying plasmids encoding an antibody are first biotinylated by the use of a commercially available biotinylation reagent (**A**). This modification is followed by the decoration of cells with the recombinant fusion protein streptavidin-ZZ (SA-ZZ) (**B**), which enables the recapturing of secreted antibodies to the cell surface (**C**).

Here we describe a novel technology for displaying full-length IgG-molecules and libraries for screening and subsequent clone characterization without requiring a genetically encoded anchor protein or intracellular antibody modification. Cell wall anchoring of secreted antibodies is achieved by chemical coupling of an Fc-binding ZZ domain that captures secreted antibodies to its surface (Fig. 1). The ZZ domain is a sequence-doubled artificial variant of protein A-derived B-domain that exhibits a high affinity to the Fc-region of various IgG-subclasses [21]. We show that antibody-secreting cells can be enriched by FACS and describe the applicability of the REAL-Select (Reversible Expression of Antibody Libraries for Selection) technology using an antibody engineering approach for affinity maturation of a phage-display derived cMet-specific antibody.

## Materials and Methods

### Plasmids

All vectors used for yeast transformation were based on the pYD1-plasmid backbone that was commercially available from Invitrogen (Yeast Display Vector Kit, version D, #V835-01). Construction of each vector was performed using the homologous recombination machinery in *S. cerevisiae* [22], [23]. Antibody genes for that purpose were amplified using the Phusion High-Fidelity DNA-Polymerase (New England Biolabs) with HPLC-purified primers (Eurofins MWG Biotech) introducing a 40 to 50 bp extension of homologous sequences at both sides. To enable selection of heavy and light chain plasmids in yeast, the light chain plasmid contained a Leu auxotrophy marker, while the heavy chain plasmid encoded a Trp-marker. VH and VL regions from each antibody to be displayed (matuzumab, adalimumab, anti-cMet-B10, trastuzumab) were cloned into the respective plasmids already containing the signal sequence and IgG1 CH1-Fc regions (pYD-mcs-CH1-Fc) or lambda/kappa contant regions. Soluble antibody secretion was directed using the αMFpp8 signal sequence [24] that was cloned in-frame 5′ of the antibody gene. The expression of the antibody genes was driven by the galactose-inducible Gal1-promoter.

### CDR-H3 library generation

A CDR-H3 mutated library containing the VH-region of an in-house selected human cMet-specific phage display derived antibody was ordered and obtained from GeneArt (Life Technologies). Within the library comprising a 30 nucleotide DNA-stretch, a doping mixture was chosen to keep each amino acid in CDR-H3 parental with a frequency of 60–70% and to avoid the introduction of stop-codons as well as cysteine and methionine residues. The synthesized dsDNA construct was used as a template for PCR amplification. During PCR a 45 bp extension for gap repair cloning in yeast to both sides of the library was achieved. 96 reactions were performed using the Phusion High-Fidelity DNA polymerase (New England Biolabs) according to the manufacturer's protocol. For each reaction 50 ng of the pre-amplified template DNA was used in a total volume of 50 µl. The reactions were combined after completing PCR and purified using the Wizard SV Gel and PCR Clean-Up system (Promega) and a final amount of 102 µg library-DNA was obtained carrying homologous sequence-attachments to the acceptor-plasmid 5′ and 3′ of the sequence. The following primers were used for amplification (up-primer 5′- CTATTGCCAGCATTGCTGCTAAAGAAGAAGGGGTACAACTCGATAAAAGAG AAGTGCAGCTGGTGCAGTCTG-3′, low-primer 5′- CTCTTGGAGGAGGGTGCCAGGGGGAA GACCGATG GGCCCTTGGTGGAGGCTGAGGAGACGGTGACCAGGG-3′) to enable the cloning of the library in-frame with the signal peptide and the human CH1-Fc region that were already included in the plasmid backbone. For gap-repair cloning the pYD-mcs-CH1-Fc was linearized using the restriction enzymes *Bam*HI and *Eco*RI and purified via Wizard SV Gel and PCR Clean-Up system. The library generation via gap repair cloning in EBY100 cells was performed following the protocol established by Benatuil and colleagues [25]. Ten electroporations were conducted with 4 µg of the linearized vector and 10 µg of the PCR-product. The library size was estimated by dilution plating and revealed 1.5×10^9^ transformants.

### Yeast strains and media

The yeast strain that harbored antibody light chains was *S. cerevisiae* strain BJ5464 (MATα URA3-52 trp1 leu2Δ1his3Δ200 pep4::HIS3 prb1Δ1.6R can1 GAL) obtained from the American Type Culture Collection (ATCC). The *S. cerevisiae* strain EBY100 (MATa URA3-52 trp1 leu2Δ1 his3Δ200 pep4::HIS3 prb1Δ1.6R can1 GAL (pIU211:URA3)) harbored antibody heavy chains and was used to generate the parsimonious heavy chain library. This strain was obtained from Invitrogen as part of the pYD1 Yeast Display Vector Kit (#V835-01, Life Technologies). The whole antibody and library was secreted by resulting diploid cells after mating.

For the cultivation of yeast cells harboring heavy and/or light chain plasmids, media containing all essential reagents except tryptophan and/or leucin was prepared using a commercially available drop-out mix and a minimal SD-base mix (#630414, #630413, #630417 and #630411, Clontech). The induction of gene expression was carried out in the same drop-out mix combined with minimal SD-base Gal/Raf (#630421, Clontech), 1 M buffer containing 8.56 g NaH_2_PO_4_ and 5.4 g Na_2_HPO_4_, pH 7.4 and 11% w/v PEG8000. Rich medium (YPD) used for yeast cell mating was prepared from 20 g glucose, 20 g peptone and 10 g yeast extract (Merck KGaA). Freezing medium was prepared using 2% glycerol and 0.67% yeast nitrogen base (BD).

### Mating

Yeast mating was used for the combination of the CDR-H3 library in haploid EBY100 cells (Mat a) with the corresponding light chain in haploid BJ5464 cells (Mat α) to gain diploid cells harboring heavy and light chain plasmids. Therefore, yeast cells carrying the plasmid for the antibody, heavy or light chain, respectively, were at first independently cultivated in their respective selective medium over night at 30°C and 250 rpm. The next day, 1×10^8^ cells of each strain were resuspended in 50 µl of YPD-medium, mixed and dripped onto the middle of a pre-warmed YPD-plate which was afterwards cultivated at 30°C over night. The thin cell layer was then washed off with 10 ml of YPD-medium. To calculate the efficiency of the mating process, the OD_600_ of the cell suspension was measured and dilution plates were prepared. The cell suspension was cultivated in 500 ml of double selective medium. 2×10^9^ cells were subsequently transferred into fresh medium after 24 and 48 hours of cultivation. Afterwards the diploid cells were resuspended in freezing medium and transferred to a cryo-vial and stored at −80°C.

### Streptavidin-ZZ (SA-ZZ) preparation

The DNA-sequence for the chimeric construct of streptavidin and *Staphylococcus aureus* protein A-derived ZZ-domain was synthesized at GeneArt (Life Technologies) and cloned into a pCMV-based vector containing the PAC selection marker. The synthesized sequence contains: a human growth hormone signal peptide, the streptavidin gene, a GS-linker and two copies of the Z-domain. CHO-S cells were transfected with this plasmid to produce the protein. The protein was then purified from the supernatant by affinity chromatography using IgG-sepharose and size exclusion chromatography (HiLoad Superdex 200 pg column, GE Healthcare).

### Cell surface manipulation

Yeast cells were biotinylated using a 3.4 kDa biotin-PEG-SCM (Creative PEGWorks). To achieve this, 1×10^7^ cells were washed twice with carbonate-buffer (4.2% NaHCO_3_ and 0.034% Na_2_CO_3_, pH 8) and resuspended in a final amount of 40 µl of the buffer containing 1–4 mg of dissolved biotin-reagent. The mixture was then incubated for 15 minutes at room temperature. The cells were pelleted and washed twice with 1 ml PBS containing 100 mM glycine to saturate free biotin-PEG-SCM. The subsequent functionalization of the biotinylated cells was performed by incubating the cells with 0.76–1.52 µM of the streptavidin-ZZ fusion in PBS on ice for another 15 minutes. In a final step, the cells were washed once with 1 ml PBS.

### IgG display, fluorescence staining and FACS

For recapture and display of secreted IgG-molecules on yeast cells the expression was performed in a static culture using petri dishes or deep-well plates for 20 hours at 20°C at an initial cell concentration of 1×10^7^ cells/ml. To specifically label surface biotin, 1×10^7^ biotinylated cells were incubated with 1.3 µM of a streptavidin-DyLight633 conjugate (Thermo Scientific/Pierce) in 20 µl at 4°C for 15 min without light and once washed with PBS after labeling. The staining of surface-immobilized ZZ domain was carried out by incubating 1×10^7^ cells with 3.3 µM in 20 µl of a protein A-specific FITC-conjugated detection antibody originating from goat (Abcam) for 15 min in the absence of light at 4°C. Following antibody incubation, the cells were washed once with PBS and kept on ice until analysis. The staining of displayed antibodies was performed using 0.5 µM in 20 µl of an AlexaFluor647-conjugated goat anti-Fc F(ab′)_2_-fragment or PE-conjugated goat anti-Fc antibody (both Jackson Immunoresearch) and different concentrations of the corresponding fluorescence labeled antigen (cMet or EGFR, Merck Serono and TNFα, R&D Systems). The labeling of the antigens cMet and EGFR was done using the LYNX rapid RPE kit (BioRad). The labeling of TNFα was done using DyLight650 NHS ester (Thermo Scientific). For the staining with PE-conjugated antigen cells were incubated on ice and in the absence of light for 15 min and washed once with 1 ml PBS. The concentration of externally given antibody golimmuab (MSD) to label free surface ZZ domains was titrated prior to the experiment to determine the maximum signal intensity. Golimumab treated cells were afterwards incubated with 250 nM of Dyligt650-conjugated TNFα. The selection of the CDR-H3 library was carried out with the MoFlo XDP cell sorter (Beckman Coulter) using Summit 5.3. In the initial selection 2×10^8^ cells were processed. During the following two rounds of sorting the remaining diversity of the library was at least 100-fold oversampled. To determine the antigen concentration for each consecutive sorting round that was used to enrich cells with enhanced affinity, the cell population in each round was stained with a set of different concentrations of the fluorescently labeled target and analysed by FACS. The lowest antigen concentration was chosen for library screening, for which compared to the control sample (no addition of fluorescent target) in the gate of 0.1% of the cells that displayed highest target-binding fluorescence a higher percentage of double stained cells was found. This gate was also used for library sorting.

### Subcloning, mammalian expression and protein purification

Subcloning of antibody genes into mammalian expression plasmids was performed to enable soluble production of IgG molecules in Expi293F cells (Life Technologies). Therefore, the gene of interest was amplified with homologous overlaps to the acceptor plasmid (Lucigen) by PCR. One Shot TOP10 chemically competent *E. coli* cells (Life Technologies) were afterwards incubated with 1 µl of the PCR-product and 1 µl of the plasmid for 30 min and transformed according to the manufacturer's protocol. The selection of clones occurred on LB-amp plates. Following incubation at 37°C for 24 hours colonies were picked, plasmid-DNA was isolated and sent to MWG Biotech for sequencing. The correct plasmid-DNA was then used for the transfection of Expi293F cells by the ExpiFectamine 293 transfection kit (Life Technologies) following the manufacturer's protocol. Cells were incubated at 37°C, 180 rpm and 5% CO_2_ for 5 days. The IgG containing supernatant was harvested by centrifugation of the cell suspension at 1500×g for 10 min. Antibodies were purified from the supernatant using PROSEP-A Spin Columns (Merck Millipore).

### Binding kinetics

Binding kinetics of subcloned and purified IgG-molecules were analyzed using the Octet RED system (ForteBio, Pall Life Science). Antibodies were captured on anti-human-FC (AHC) biosensors for 600 s at 2.5 µg/ml in PBS. All measurements were performed in kinetics buffer (PBS pH 7.4, 0.1% (w/v) BSA, 0.02% Tween 20). Association to cMet (10 nM, 50 nM, 100 nM) was measured for 300 s followed by dissociation for 600 s. One control of each antibody was analyzed using kinetics buffer only and subtracted from all binding curves resulting from the interaction with cMet. Processed binding curves were evaluated with the ForteBio data analysis software 8.0 by using a 1∶1 binding model after Savitzky–Golay filtering.

## Results

### Cell surface functionalization for antibody capture

**Figure 2:**
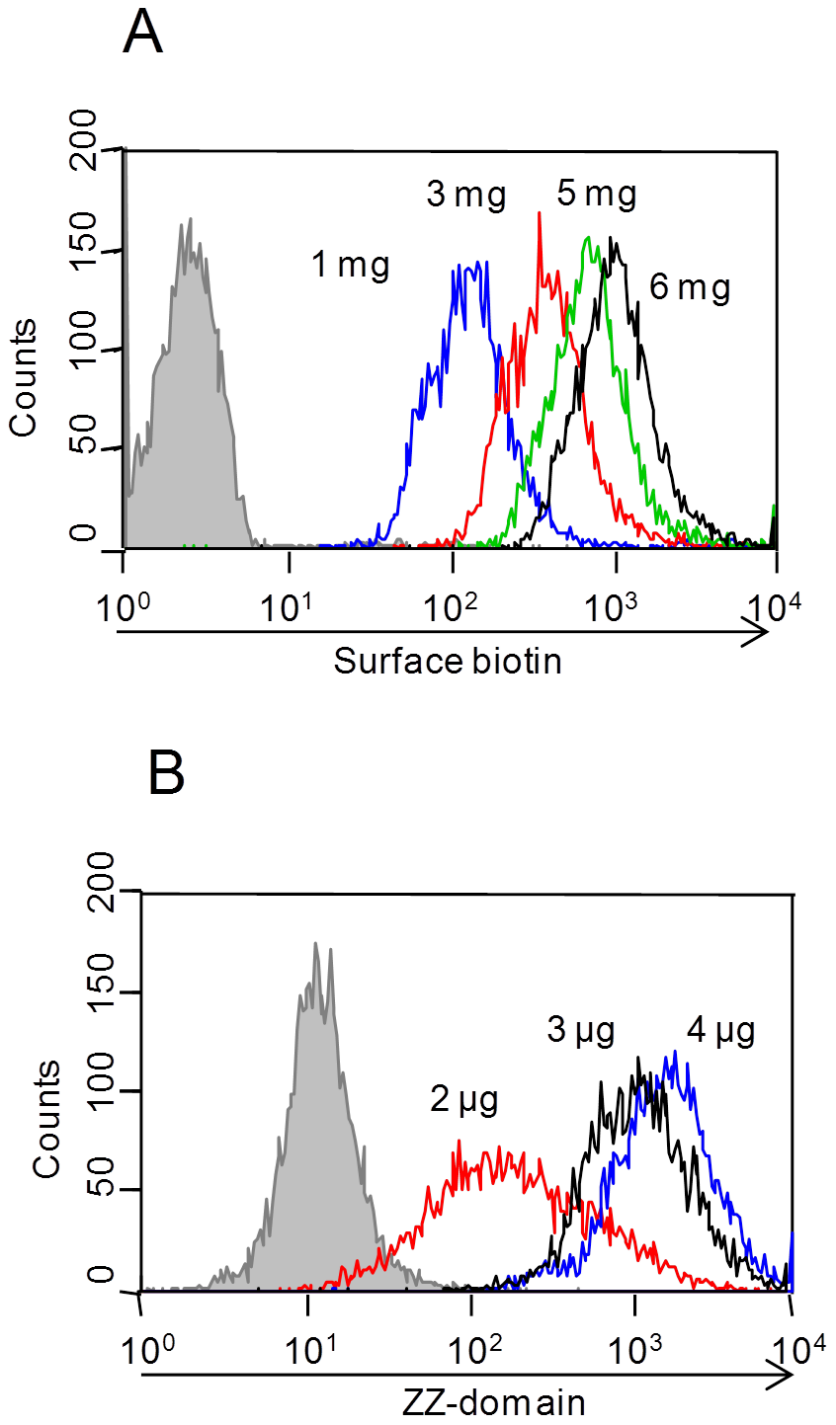
Titration of reagents for biotin-labeling and SA-ZZ immobilization on yeast cells. Respective indirect fluorescence signals were analyzed by flow cytometry. (**A**) 1×10^7^ cells were labeled with 1 mg to 6 mg biotin reagent and stained with streptavidin-Dylight633. (**B**) 1×10^7^ cells labeled with 1 mg biotin were incubated with 2, 3 or 4 µg of SA-ZZ for immobilization of the Fc-capture moiety. Subsequently surface ZZ domain was detected using a FITC-conjugated goat-anti-protein A antibody.

For antibody capture, a fusion protein consisting of streptavidin and a ZZ domain (SA-ZZ) was constructed and purified from stably transfected CHO-S cells (kindly provided from Inter-Lab, Israel). To investigate whether the fusion can be captured to the cell surface while maintaining the antibody-binding capability, the cell surface was biotinylated using a commercial available biotin-reagent followed by addition of the recombinant SA-ZZ fusion protein. Initially, amounts of biotin-reagent and SA-ZZ were tested aiming for sufficient labeling of the cell surface. For that purpose, BJ5464 *S. cerevisiae* cells were biotinylated using 1 – 6 mg of biotin-PEG-SCM 3.4 kDa per 1×10^7^ cells. To analyze the extent of surface label, cells were incubated with streptavidin-Dylight633 and the fluorescence was detected by flow cytometry. With increasing amounts of the reagent the fluorescence-signal enhanced continuously (Fig. 2A). As cells that are labeled with 1 mg of the biotin reagent (blue) already showed an increase in fluorescence by about one order of magnitude compared to the negative control (grey), 1 mg reagent was chosen for subsequent SA-ZZ immobilization to avoid avidity effects upon antigen binding that may arise upon high density antibody loading onto the cell surface. Different quantities of SA-ZZ (2 µg, 3 µg, 4 µg) were used for the incubation with biotinylated cells to functionalize the cell surface with the IgG-capture domain. Functionalized cells were incubated with a protein A-specific FITC-conjugated goat antibody allowing flow cytometric analysis of cell surface-immobilized ZZ. Increasing the amount of SA-ZZ resulted in an enhancement of the fluorescence signal (Fig. 2B), caused by the higher surface density of immobilized capture-domains. A distinct fluorescence shift compared to the negative control (grey) was detected for all SA-ZZ concentrations tested.

### IgG-capture

**Figure 3:**
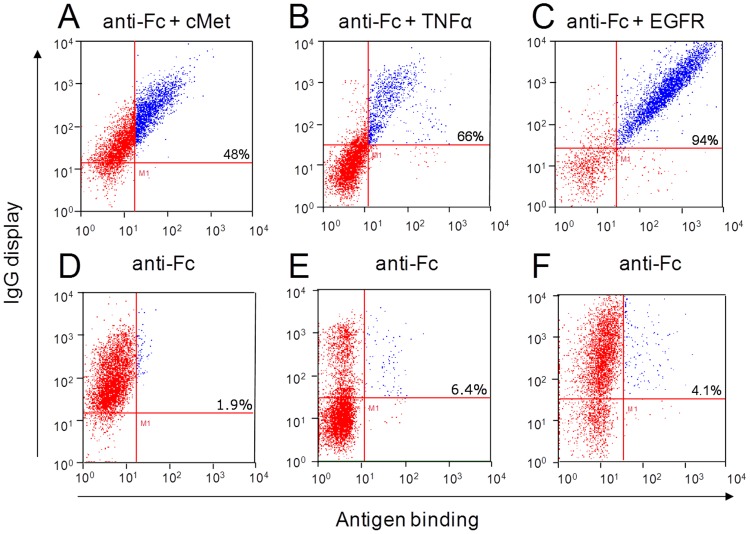
REAL-Select functionalized yeast cell phenotype of three different mAbs analyzed by flow cytometry. (**A**) anti-cMet-B10, (**B**) adalimumab (**C**) matuzumab and the respective controls without (**D**) cMet, (**E**) TNFα, (**F**) EGFR. Display of functional antibodies was detected using the respective fluorescence-labeled antigen (**Table 1**) and an Fc-specific detection antibody (IgG display). The percentage indicated in the upper right gate represents the percentage of cells in that gate, normalized to the total number of antibody displaying cells (sum of the cells in the upper left and right gate).

To investigate the ability of functionalized cells to still secrete soluble high-quality antibodies, BJ5464 cells were transformed with heavy and light chain plasmids encoding three different monoclonal antibodies. These antibodies were matuzumab (K_D_ 0.34 nM [26]), adalimumab (K_D_ 30 pM [27]) and anti-cMet-B10 (analyzed with K_D_ 40 nM) (Table 1). Analysis of IgG-capture was carried out by incubation of the modified cells with the fluorescently labeled antigen listed in Table 1 and an anti-Fc specific AlexaFluor647-conjugated F(ab′)_2_ fragment or an goat anti-Fc PE-conjugated antibody for detection of IgG display by flow cytometry (Fig. 3A–C). As a negative control cells were only stained for the detection of IgG-display (Fig. 3D–F). The three tested double-stained cell samples showed a positive signal for antibody display and antigen binding, indicated by a double positive fluorescence signal compared to the respective negative control labeled with anti-Fc only. We note that a varying fraction of cells is present, for which no IgG secretion and display can be observed. This finding is not related to the ZZ-capture procedure since it is also observed upon Fab display via Aga2p fusion (data not shown).

**Table 1:** Antigen specificities and KD-values of antibodies displayed on BJ5464 using REAL-Select.

Antibody	Antigen	KD
Matuzumab	huEGFR	0.34 nM
Adalimumab	huTNFα	30 pM
Anti-cMetB10	hucMet	40 nM

**Figure 4:**
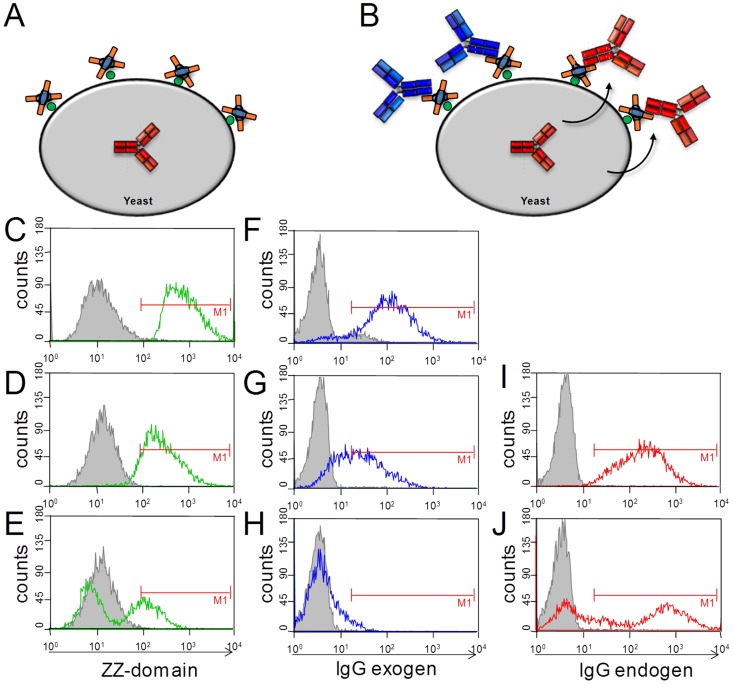
Flow cytometric analysis of capture domain saturation of REAL-Select functionalized yeast cells. (**A**) SA-ZZ decorated cells carrying matuzumab heavy and light chain plasmids, (**B**) surface capture of matuzumab (red antibody) or externally added golimumab (blue antibody). (**C–E**) Analysis of yeast cells decorated with SA-ZZ fusion protein with goat-anti-protein A-FITC 0, 6 hours and 20 hours after surface decoration and induction of matuzumab expression. (**F–H**) Detection of unoccupied Fc-capture domains by incubation of cells with golimumab followed by labeling with TNFα-Dylight650 at indicated time points. (**I,J**) Monitoring of surface display of re-captured matuzumab by labeling cells with goat-anti-Fc F(ab′)_2_-AlexaFluor647 at 6 hours and 20 hours of expression.

For further analysis of IgG expression and extent of ZZ domain occupation, BJ5464 cells carrying heavy and light chain plasmids for matuzumab were functionalized by REAL-Select and antibody expression was induced using galactose media. After cell labeling with the ZZ domain cells were allowed to grow and secrete matuzumab. As expected, the number of ZZ domains covalently attached to the cell wall decreased 6 and 20 hours after immobilization, most likely due to cell growth but degradation or inactivation of the fusion protein upon prolonged incubation in media at 20°C can also contribute to this (Fig. 4C–E). The occupation of the immobilized ZZ domains with matuzumab (Fig. 4B, red IgG) was simultaneously monitored using an goat anti-Fc PE-conjugated antibody (Fig. 4I,J) showing a similar labeling pattern as for the ZZ domain. To investigate whether unoccupied ZZ domains reside on the cell surface that are not covered by secreted matuzumab, cells were collected at three time points after induction of matuzumab secretion and incubated in excess with golimumab antibody. Golimumab binding to unoccupied ZZ domains on the yeast cell surface was monitored by addition of the corresponding fluorescently labeled antigen, TNFα. After 6 hours of expression, all functionalized cells captured endogenous matuzumab (Fig. 4I), while cell staining with the externally added antibody was low and completely absent after 20 h incubation (Fig. 4H), indicating that the ZZ domains residing on the yeast cell surface were fully saturated with endogenously produced antibody.

### Genotype-phenotype coupling and library screening

**Figure 5:**
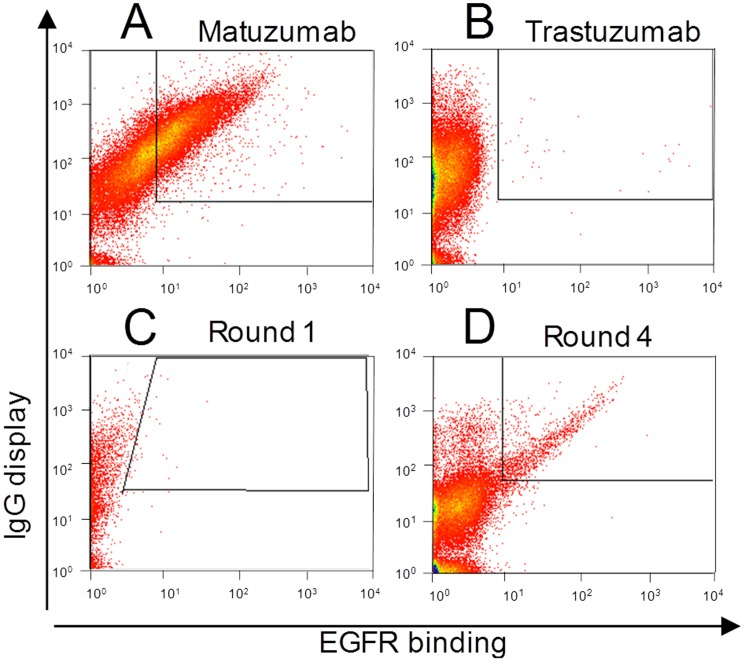
FACS analysis of REAL-Select enrichment of matuzumab-displaying cells. Functionalized yeast cells displaying (**A**) matuzumab or (**B**) trastuzumab were mixed 1∶1,000,000 (**C**) and labeled using EGFR-PE and goat-anti-Fc F(ab′)_2_ AlexaFluor647. (**C,D**) FACS analysis of the cell population of sorting round 1 (**C**) and 4 (**D**) of FACS enrichment of matuzumab displaying cells.

To verify that a genotype-phenotype linkage exists for REAL-Select, a mixing experiment was performed. EGFR-binding matuzumab displaying cells (Fig. 5A) were mixed with trastuzumab displaying cells (Fig. 5B) at a 1 to 1,000,000 ratio, mimicking the size of a conventional immune library. SA-ZZ was immobilized on the surface of yeast cells, and the mixture was transferred to induction media, incubated for 20 hours and subsequently fluorescently labeled with an EGFR-phycoerythrin (EGFR-PE) conjugate and an anti-Fc AlexaFluor647-conjugated antibody and subjected to 4 consecutive rounds of sorting (Fig. 5C,D). Sorting gates were defined using control cells not secreting/displaying an antibody and stained with anti-Fc and the antigen aimed at setting a collecting window for cells that secreted and displayed a functional antibody. After sorting and re-sorting round 4, a single cell analysis of cells for EGFR binding was performed by flow cytometry. Of ten analyzed clones, nine displayed binding to EGFR-PE, confirming successful enrichment (data not shown).

**Figure 6:**
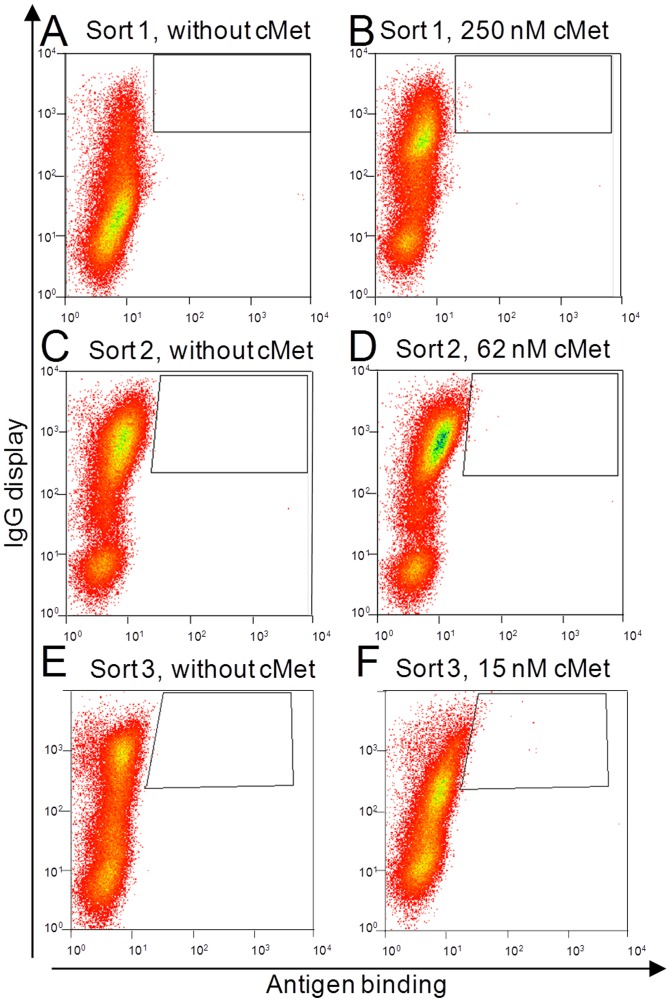
CDR-H3 library yeast cell phenotype and selection strategy for the affinity maturation of a cMet specific antibody towards three rounds of FACS screening with decreasing antigen-concentrations (B) 250 nM, (D) 62 nM, (F) 15 nM cMet and anti-Fc AlexaFluor647 for display detection. As a specific control for each sorting round, enriched library cells were induced for IgG secretion and labeled with an anti-Fc AlexaFluor647-conjugated antibody only (**A,C,E**).

Encouraged by this result, an affinity maturation of a cMet specific antibody derived from an in-house phage display library screening campaign (K_D_ 40 nM) was performed. To this end, a parsimonious mutagenesis of the CDR-H3 loop of the variable domain of the heavy chain was performed, where all ten residues were randomized with all 19 amino acids except Cys and Met, keeping the original amino acid at each position with a frequency of approximately 60–70%. The library was constructed in *S*. *cerevisiae* strain EBY100 resulting in approximately 1.5×10^9^ transformants. Sequence analysis of randomly picked clones revealed on average 3.4 substitutions within the ten residues of the CDR-H3. The haploid library cells were mated with haploid BJ5464 (Mat α) cells carrying the parental light chain of the antibody with a mating efficiency of 15%. Diplonts were selected by their ability to grow on double-selective media and decorated with SA-ZZ. The secreted antibodies were recaptured to the cell surface and labeled with an anti-Fc AlexaFluor647-conjugated F(ab′)_2_ and PE-conjugated cMet and double stained cells were isolated by FACS (Fig. 6).

**Figure 7:**
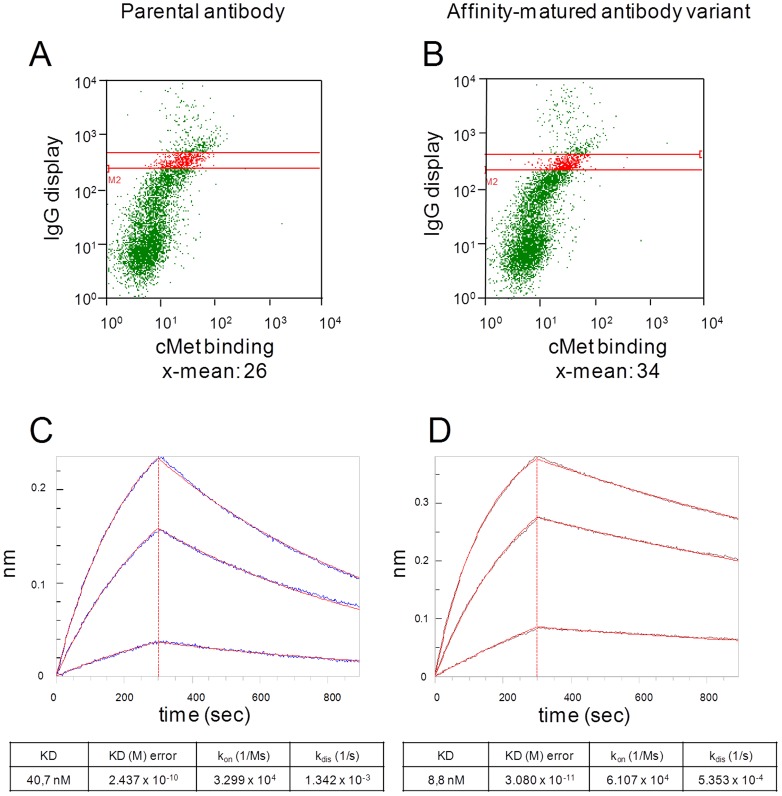
Functional characterization of an antibody with improved binding of cMet. (**A,B**) FACS analysis of (**A**) yeast cells displaying parental antibody, (**B**) cells displaying the selected antibody labeled with 100 nM cMet-PE and anti-Fc AlexaFluor647. (**C**) Binding kinetics of Expi293F expressed parental antibody and (**D**) affinity-matured antibody variant to cMet (100 nM, 50 nM, 10 nM) determined by biolayer interferometry. Association rate constants (k_on_), dissociation rate constants (k_dis_) and binding affinities (K_D_) of both antibodies were determined assuming a 1∶1 binding model.

To obtain binders with higher affinity, the cMet concentration was successively reduced from 250 nM to 15 nM over three sorting rounds. After the third round of sorting, plasmid DNA of yeast cells was isolated and used to transform *E. coli* cells. All sequences were unique, but in 5 clones Thr#7 was replaced by Ile/Leu. 11 clones were used to retransform yeast cells with the parental light chain plasmid by electroporation. A phenotypical analysis regarding cMet-binding was conducted on the surface of yeast cells. At 100 nM cMet concentration one variant exhibited a slightly enhanced fluorescence signal for the antigen binding compared to the parental antibody upon normalization of the display ratio (Fig. 7).

The selected sequence was subsequently subcloned into a mammalian vector for soluble expression in Expi293F cells. Following antibody expression and purification, kinetic analysis of the antibody variant revealed a 5-fold improved affinity to cMet (Fig. 7D) compared to the parental antibody (Fig. 7C).

## Discussion

Today in both academia and pharmaceutical companies tremendous resources are deployed to discover and develop monoclonal antibodies for target-based therapies. Among others, high-throughput techniques for screening antibody libraries enable the identification of new candidate molecules and the fast optimization of pre-selected binders by affinity maturation. In this regard, the invention of new powerful screening technologies has proven to be one critical part in an overall strategy to further accelerate the process of antibody discovery and development. Several *in vivo* display technologies have emerged since the invention of phage display in the mid-eighties [28] and the application for antibody display [29]. Yeast display is a robust technology to select and engineer antibody-fragments from combinatorial libraries. The availability of the eukaryotic secretion pathway leads to a superior expression of complex molecules compared to a bacterial host, where the expression of oligomeric molecules like full-length IgGs is often impaired [6]. In this paper we showed that the REAL-Select technology allows us to non-covalently display functional full-length IgG molecules in their native format, without sequence modifications and independent from the antibody specificity which enables the screening of diverse antibody libraries.

The method relies on the yeast cell surface attachment of a protein A-derived ZZ domain that acts as a capturing agent for the antibody. Protein A has been established as robust biotechnology tools for antibody purification over the years [30]. It was also successfully used for the inner membrane capture of full length IgG upon periplasmatic expression in *Escherichia coli* as well as for IgG display on filamentous phage [31], [32]. The Z domain is a stability-engineered analog of protein A-derived B domain [21]. The divalent ZZ domain is a sequence duplication of monovalent Z domain exhibiting a higher affinity for human Fc-fragments [33]. Based on Biacore results conducted by Nilsson and colleagues in 1994 the K_D_ of monovalent Z domain for human Fc is calculated at 50 nM, while the divalent ZZ domain shows an affinity for human Fc that is about 17-fold increased (K_D_ 3 nM [34]). The higher affinity of the ZZ for Fc is mainly driven by an 8-fold improvement in the dissociation rate [34]. Since both the ZZ domain and the bound antibody are dimers, avidity effects drive a very tight non-covalent ligand-antibody interaction and strong phenotype-genotype coupling, thereby allowing for the screening of large combinatorial libraries. Regarding antigen binding, this allows the displayed antibody to be optimally orientated on the surface of cells.

For ZZ-immobilization the streptavidin-part of the chimeric SA-ZZ construct is bound to biotin-labeled cells. Minimal concentrations of both reagents were experimentally determined. Due to the control of the externally applied reagents it was possible to exactly regulate the amount of surface immobilized capture-domain and to regulate quantity, density and surface-signal of captured antibodies. In particular for the screening of high affinity binders to bivalent or multivalent targets it may be desirable to keep the number of surface-exposed antibodies low aimed at avoiding avidity effects. In this respect, it is important to note that the number of surface exposed antibodies is comparable to or even lower than the display level of Fabs using classical YSD via chain linkage to Aga2p (data not shown).

ZZ domain-mediated antibody capture eliminates the need to modify the antibody, which is required for IgG-display in the comparable SECANT technology. In that technology the antibody is expressed as a fusion to the biotin-acceptor peptide [20] and co-expression of a biotin ligase is required to obtain secreted biotinylated antibodies that are captured by surface-conjugated avidin. Here, the antibody is displayed in its final and native format which may contributes to its overall performance during library selection and clone characterization in the screening process. In contrast to the SECANT technology, there is no need for the co-expression of additional factors like the biotin ligase, which gives the freedom to choose any appropriate expression host. One major advantage lies in the fact that one can easily switch between display and secretion mode, thereby speeding up process cycle-times during antibody discovery and characterization of single candidate proteins.

Since genotype-phenotype linkage is thought to be a possible liability within the REAL-Select technology, a saturation experiment was performed to figure out the optimal duration for antibody expression in terms of ZZ-occupation. If only a minor amount of surface immobilized ZZ domains are occupied by endogenously secreted IgG-molecules there might be a risk that free capture domains bind antibodies secreted from foreign cells thus preventing a reliable phenotypical enrichment of library clones. We found that nearly all immobilized ZZ domains are occupied by secreted antibody molecules within 20 hours of expression and already after 6 hours a sufficient amount of antibody was displayed on the cell surface (Fig. 4). Also within the first 6 hours of expression, the amount of immobilized ZZ domain seems to be stable. A second non-functional cell population emerged after 20 hours indicating cell budding. This new cell generation is not functionalized with capture domain and therefore does not contribute to the display of variants. Nevertheless, we observed an increase in fluorescence for IgG-display of the functionalized population which is indicative of the saturation of capture domains (Fig. 4I,J).

The first proof of a stable genotype-phenotype linkage was given within a mixing experiment. In this experimental set-up matuzumab displaying cells were highly diluted in trastuzumab displaying cells and through antigen-mediated fluorescence matuzumab cells were recovered from the mixture during four FACS-rounds. The final evidence for a stable genotype-phenotype linkage was demonstrated using the CDR-H3 parsimonious library for affinity maturation as an example of applicability of REAL-Select. We chose a parental antibody with an initial relatively high affinity to human cMet (K_D_ 40 nM) to investigate whether subtle but significant improvements in affinity can be detected upon affinity maturation. A CDR-H3 library of the antibody heavy chain was generated in EBY100 cells (Mat a). Due to library design a theoretical diversity of about 1×10^13^ was calculated with the highest probability of variants having 3 to 4 mutations at the amino acid level. In general, the variable loop of CDR-H3 is known to comprise a large diversity in the human antibody repertoire and residues of CDR-H3 are strongly involved in antigen contact [35]. A library size of 1.5×10^9^ yeast transformants was by far insufficient to cover the theoretical diversity of the parsimonious approach, but was already at the limit for comprehensive screening by FACS. In the model experiment shown here, in the initial library only a small subset of clones retained the ability to bind cMet, and binders that retained antigen binding could be enriched over three screening rounds. The variant with improved affinity (5-fold) displayed a single amino acid exchange (Thr → Leu) in CDR3, despite the fact that the average mutation frequency was much higher. One could argue that in this non-covalent system it would be impossible to select for higher affinities than the affinity of ZZ to Fc. However, it should be taken into consideration that due to the (adjustable) high surface density of immobilized ZZ-domains it can be expected that not only affinity but also avidity effects contribute to efficient ZZ-Fc binding, eventually contributing to strong genotype-phenotype coupling.

In conclusion, the technology described here has been proven to be suitable for antibody library screening using a non-covalent surface attachment of an antibody. To our knowledge it is the first time that full-length IgG-molecules were successfully displayed on *S. cerevisiae* cells in an unmodified format for the purpose of library screening. A further advantage to such non-covalent display is the possibility to selectively switch between display mode and soluble production of the antibody. Since the antibody capturing moiety is placed on the cell surface by chemical conjugation rather than genetic encoding, no restriction for the use of the host strain exists and any expression host that has been optimized for antibody expression can be used. In this regard CHO cells are currently the standard host for industrial antibody production. Hence, application of REAL-Select in CHO cells provides the opportunity to immediately interrogate the consequence of antibody affinity or stability maturation on expression yield using any production host. As a consequence, this approach allows one to considerably accelerate the screening process by omission of sub-cloning steps and host changes that are inevitable within other surface display technologies.
